# The influence of visuospatial attention on unattended auditory 40 Hz responses

**DOI:** 10.3389/fnhum.2013.00370

**Published:** 2013-07-15

**Authors:** Cullen Roth, Cota Navin Gupta, Sergey M. Plis, Eswar Damaraju, Siddharth Khullar, Vince D. Calhoun, David A. Bridwell

**Affiliations:** ^1^Department of Mathematics and Statistics, University of New MexicoAlbuquerque, NM, USA; ^2^Department of Biology, Initiative for Maximizing Student Development, University of New MexicoAlbuquerque, NM, USA; ^3^The Mind Research Network, University of New MexicoAlbuquerque, NM, USA; ^4^Chester F. Carlson Center for Imaging Science, Rochester Institute of TechnologyRochester, NY, USA; ^5^Department of Electrical and Computer Engineering, University of New MexicoAlbuquerque, NM, USA

**Keywords:** EEG, 40 Hz, gamma band, SSAEP, frequency tagging, ICA, tetris, visuospatial attention

## Abstract

Information must integrate from multiple brain areas in healthy cognition and perception. The present study examined the extent to which cortical responses within one sensory modality are modulated by a complex task conducted within another sensory modality. Electroencephalographic (EEG) responses were measured to a 40 Hz auditory stimulus while individuals attended to modulations in the amplitude of the 40 Hz stimulus, and as a function of the difficulty of the popular computer game Tetris. The steady-state response to the 40 Hz stimulus was isolated by Fourier analysis of the EEG. The response at the stimulus frequency was normalized by the response within the surrounding frequencies, generating the signal-to-noise ratio (SNR). Seven out of eight individuals demonstrate a monotonic increase in the log SNR of the 40 Hz responses going from the difficult visuospatial task to the easy visuospatial task to attending to the auditory stimuli. This pattern is represented statistically by a One-Way ANOVA, indicating significant differences in log SNR across the three tasks. The sensitivity of 40 Hz auditory responses to the visuospatial load was further demonstrated by a significant correlation between log SNR and the difficulty (i.e., speed) of the Tetris task. Thus, the results demonstrate that 40 Hz auditory cortical responses are influenced by an individual's goal-directed attention to the stimulus, and by the degree of difficulty of a complex visuospatial task.

Multiple sensory areas must integrate across the brain in order to facilitate healthy cognition and behavior. Integrating information over multiple modalities may be beneficial in some instances but disadvantageous in others. For example, information from both the auditory and visual cortex provides useful information when listening to a speaker, since each modality facilitates understanding the speaker (Macaluso et al., 2004). Alternatively, complex tasks such as reading depend upon enhanced processing within visuocognitive areas that support reading, while placing less emphasis on the processing of irrelevant senses (e.g., auditory responses to individuals talking nearby) (Welcome and Joanisse, 2012).

Within a single modality, attending to a particular aspect of a feature results in an enhanced response toward that feature and a reduced response toward unrelated aspects of that feature. For example, attending to a direction of motion results in enhanced responses within visual areas sensitive to the attended direction, and reduced responses within visual areas sensitive to the orthogonal direction of motion (Treue and Martinez-Trujillo, 1999; Saenz et al., 2002). The enhancement and suppression of relevant and irrelevant aspects of a feature has been widely demonstrated within a single modality, such as vision, but they appear less prominent across modalities (Talsma et al., 2006). In particular, the degree in which attention toward one modality (e.g., vision) modulates attention to another modality (e.g., audition) appears to depend on the difficulty and complexity of the attended task and the location in which unattended responses are measured (Hein et al., 2007).

A contributing factor to the degree in which auditory and visual tasks compete may be the degree of overlap between the brain areas involved in each task. Hein et al. (2007) demonstrate that frontal, parietal, and middle temporal responses partially overlap between simple visual detection and auditory detection. These overlapping activations indicate common brain areas involved in the perceptual and behavioral response to visual or auditory stimuli. In agreement with this suggestion, the authors show that detecting an auditory target diminishes the subsequent response to visual targets within frontal and middle temporal cortex, as well as within early visual areas. These findings are in qualitative agreement with a monotonic decrease in transient 40 Hz auditory responses when individuals attend to auditory tones, when they are passive, and when they engadge in reading (Tiitinen et al., 1993). In addition, the auditory-evoked response has been demonstrated to be sensitive to visuomotor load (Yucel et al., 2005; Haroush et al., 2010), and the response to emotional auditory stimuli is diminished with increasing visual load (Mothes-Lasch et al., 2012). Other studies have failed to demonstrate this effect (Parks et al., 2011), or have demonstrated the opposite pattern of results (Regenbogen et al., 2012).

In the following experiment, we examine the modulation of auditory 40 Hz EEG responses [i.e., steady state auditory-evoked potential's (SSAEP's)] with attention to the auditory stimuli, and as a function of the difficulty of the popular computer game Tetris. Tetris is a complex visuospatial task which requires visual detection and spatial rotation of the Tetris pieces. The task incorporates sudden visuospatial judgments with the appropriate motor movements to rotate the pieces and move them horizontally along the screen (Sims and Mayer, 2002). The requirement for attentional and perceptual resources within the task provides a promising avenue for testing the influence of attentional and perceptual load on irrelevant sensory processing (Lavie, 2005). We hypothesize that increased task load (e.g., a higher level of difficulty in the game Tetris) will be associated with enhanced recruitment of brain areas toward the visuospatial task and reduced recruitment of areas involved in processing or integrating the unattended auditory input. For further comparison, the magnitude of auditory responses during the Tetris task was compared to the magnitude of responses when individuals attend to the auditory stimuli directly.

## Materials and methods

### Participants

Nine individuals (all males) between the ages of 21 and 40 volunteered to participate in two sessions consisting of a reference session and a SSAEP session. One individual was excluded from analysis due to experimental error, bringing the total number of subjects to eight. Each individual had normal or corrected-to-normal vision and had no family history of mental illness. Written informed consent was obtained prior to the first session.

### Design and stimuli

All experimental stimuli were produced and presented on a computer using MATLAB (The MathWorks). A Tetris task was displayed within a figure (6.5° by 12.5° visual angle) in the middle of the screen. The squares that comprised the Tetris pieces were 0.6° by 0.6°. Individuals pressed the left and right arrow keys to move the pieces left and right, respectively, and the spacebar to rotate the pieces counterclockwise. The down arrow key was disabled to ensure that individuals could not advance the piece further down the screen on their own, since this would create variability in the speed of the pieces and the difficulty of the task. Each completed row was worth 10 points regardless of whether one or multiple rows were completed at once in order to encourage individuals to complete rows as quickly as possible. The score was presented to the individual at the top of the screen for the duration of the 5 min trial. The task restarted if the individual received a “game over” before the trial was complete, although the scores were carried over.

**Figure 1:**
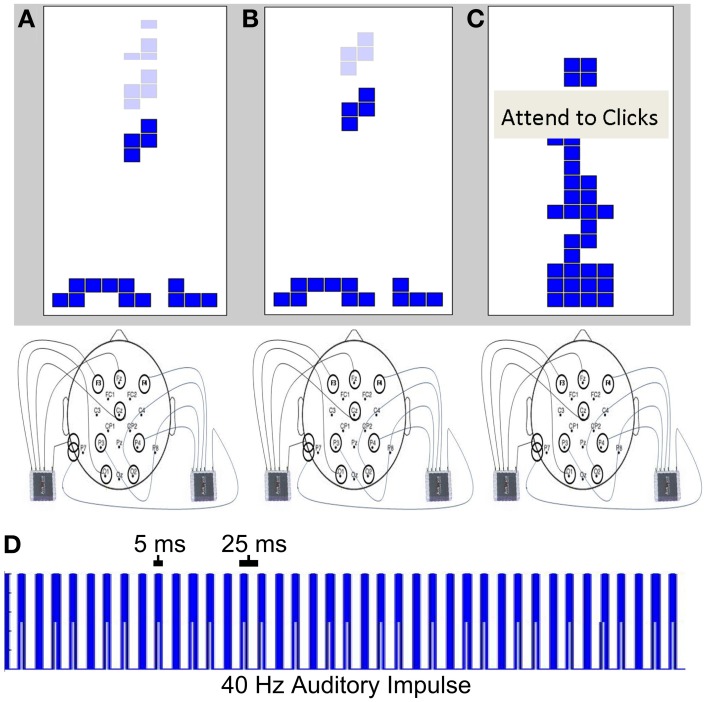
**SSAEP conditions**. Individuals participated in a difficult version of Tetris (**A**, condition 1), an easy version of Tetris (**B**, condition 2), or attended to the auditory stimulus (**C**, condition 3). A 40 Hz auditory stimulus was presented to the subject during each condition, generating a 40 Hz SSAEP response. The stimulus consisted of a series of 5 ms square waves with a 25 ms interval between the onset of each pulse **(D)**.

The 40 Hz auditory stimulus consisted of a series of 5 ms square waves with a 20 ms pause between each square wave (Figure 1D). The intensity of the auditory stimuli were modulated randomly by reducing the amplitude of 1% of the square waves by 75% (selected at random). These modulations were inserted within the auditory stimuli during all EEG recordings in order to enhance the salience of the auditory stimuli. Individuals were instructed to attend to these modulations when they attended to the auditory stimulus in condition 3. The auditory stimulus was presented to the subject continuously throughout the EEG session via headphones (70 dB; binaural; sampling rate = 44,100 Hz).

### Tetris reference session

Each individual participated in an initial session to measure the level of Tetris in which they have peak performance. This reference session consisted of two blocks of the task. Each block consisted of 5 levels of Tetris (5 min. per level) in ascending levels of difficulty. The difficulty of each level was set by adjusting the duration before the piece advanced 0.6° toward the bottom of the figure. The following 5 levels were used: 255, 181, 138, 94, 50 ms. These levels correspond to movements in the Tetris pieces at approximate frequencies of 3.92, 5.52, 7.25, 10.64, and 20 Hz. The levels were chosen based upon initial pilot testing, which indicated that they capture the range of difficulties for both novice and experienced Tetris players.

Individuals took a brief break after the first 25 min block of 5 trials, then participated in a second identical block. The scores from the two blocks were averaged together and the level with the maximum score was used as the reference level for the speed of the Tetris pieces for that individual in the SSAEP session that followed.

### SSAEP session

The SSAEP session began with an initial 5 min practice trial to allow individuals to become reacquainted with the Tetris controls. Individuals were instructed that they would either play Tetris or fixate on Tetris while attending to the auditory stimulus. The 40 Hz auditory stimulus was presented to the subjects throughout the session binaurally with headphones and 40 Hz cortical responses were recorded with EEG (see SSAEP recording and analysis, below). The level of difficulty of Tetris was adjusted by subtracting the individual subjects reference level from the first session by 50 ms (condition 1; difficult, Figure 1A) or by adding 200 ms to their reference level (condition 2: easy, Figure 1B). (Note that 50 ms was not subtracted from the subject with the reference level of 50 ms, since it was below the threshold of possible values). The words “Attend Clicks” were displayed over the Tetris game to instruct participants to attend to the modulations of the auditory stimuli (condition 3, Figure 1C). During this condition, individuals remained fixated at the center of the screen while the Tetris pieces fell, without rotating, at the same speed as in condition 1 (i.e., the subjects reference level minus 50 ms) (e.g., as depicted in Figure 1C). The keyboard controls were disabled and individuals were instructed to attend to the subtle variations in the amplitude of the 40 Hz stimulus. Individuals participated in each of the three conditions three times within a single experimental session over the 45 min session (e.g., 5 min per condition × 3 conditions × 3 repeats per condition). The order of conditions was determined for each individual using a latin square design. The latin square consisted of a 3 × 3 matrix with the elements 1, 2, and 3 such that no element repeated within a given row or column. The rows of the square were randomly drawn for each individual to create a 9 element vector of conditions within a given session. This ensures that individuals never participated in the same condition consecutively, and that the order of conditions was randomized across individuals.

### SSAEP recording and analysis

EEG responses were collected with two 4 channel CamNtech Actiwave™ mobile EEG devices (sampling rate: 256 Hz). The eight electrodes where placed on the subject's scalp at the 10–20 locations F3, Fz, F4, Cz, P3, P4, O1, and O2, with a left mastoid reference. EEG analysis was conducted in MATLAB using custom functions, built-in functions, the Statistics Toolbox, and EEGLAB (Delorme and Makeig, 2004). Temporal independent component analysis (ICA) was conducted on each individual's detrended bandpass filtered (~2–50 Hz) EEG data [extended Infomax, (Bell and Sejnowski, 1995; Lee et al., 1999)].

The spectral amplitude of the ICA sources were calculated within 2 s windows with 1 s overlap, generating 298 epochs for each 5 min trial. The epochs were averaged across intervals and conditions. The log amplitude of the spectral response at 40 Hz was normalized by the average log amplitude at 38 and 42 Hz, generating the SNR. This measure ensures that the 40 Hz responses are isolated to the auditory stimulus and attenuates the possibility that broadband muscle artifacts influence the observed results.

ICA sources were isolated if the log SNR of the 40 Hz response exceeded 0.3. This threshold was chosen since curves that exceed that value demonstrate a robust peak visually at 40 Hz. The isolated ICA sources were reconstructed to the original data space. The log SNR was calculated separately for each condition by averaging across each epoch [298 epochs per trial × 3 trials = 894 epochs] for each condition, separately for each subject. There was variability in the spatial location of the peak response across subjects, potentially due to the use of a left mastoid reference. Thus, subsequent analysis was conducted on the channel with the largest SNR within each subject. It is important to note that these ICA exclusion and channel selection criteria were unbiased with respect to differences across the conditions since the SNR values were examined after collapsing across all conditions.

Our goal was to determine whether cortical responses to an auditory stimulus are modulated by attention to the stimulus, and by the degree in which individuals attend to a complex visuospatial task. Differences in the response to the auditory stimuli were examined across conditions using a within subjects One-Way ANOVA with factor “condition” and log SNR as the dependent variable. The correlation between 40 Hz responses and the speed of the Tetris pieces was examined in order to further determine whether reduced auditory responses are associated with increased task speed (i.e., difficulty).

## Results

### Behavioral performance

Tetris scores were collected for each individual at the end of each 5 min game. Two scores were collected for each level of difficulty and the two scores were averaged to determine the level in which each individual had the highest score. Four individuals demonstrated the highest score at speeds of 7.25 Hz, while the remaining four individuals had peak scores at 3.92, 5.52, 10.63, and 20 Hz. These levels served as a reference for the level of difficulty within the SSAEP experiment that followed. During the SSAEP session, an average of 4.55 lines and 2.05 lines were completed per minute for the difficult and the easy task, respectively.

### Independent component analysis

**Figure 2:**
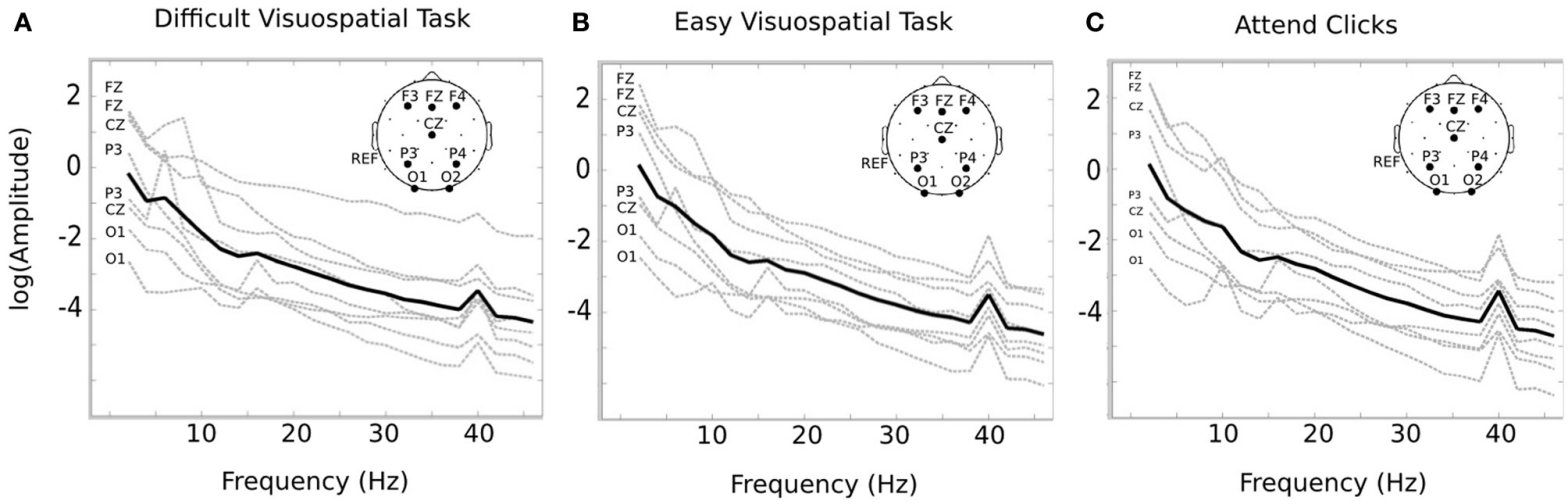
**Average SSAEP response for each condition**. The spectral response is displayed for each subject for the difficult visuospatial task **(A)**, the easy visuospatial task **(B)**, and for “attend clicks” **(C)**. The spectral response was obtained from the channel with the largest response at 40 Hz after ICA reconstruction (the channel is indicated on the left of each plot). The log amplitude of the response is shown for each subject in gray, and the overall average is in black. Each individual demonstrates a response at 40 Hz, which corresponds to the frequency of the auditory stimulus. The topographic image on the upper right of each plot indicates the 10–20 locations for F3, Fz, F4, Cz, P3, P4, O1, and O2 (left mastoid reference).

Temporal ICA was conducted on the EEG time course from session 2 in order to isolate temporal sources that respond to the stimulus frequency. The amplitude spectra of the temporal components indicate that each subject demonstrated a peak at 40 Hz within at least one source. In general, we observed the highest response within frontal electrodes. For example, the ICA source with the largest SNR contained the highest loading over Fz (1 subject), F3 (3 subjects), F4 (1 subject), P4 (2 subjects), or O1 (1 subject). The temporal components were selected if they had a log SNR greater than 0.3, and reconstructed into channel space. The average number of components identified was 3.125 (max = 6; min = 1). After reconstructing to channel space, the channel with the largest SNR was chosen for further analysis. Different channels were used for each individual since there was variability in the location of the peak response across individuals. Figure 2 indicates the log amplitude for the channel with the peak response in each subject from 2 to 42 Hz. Each of the 8 individuals demonstrates a relatively smooth decline in the spectral response with a clear peak at the stimulus frequency of 40 Hz (Figure 2).

### Attention and auditory responses

**Figure 3:**
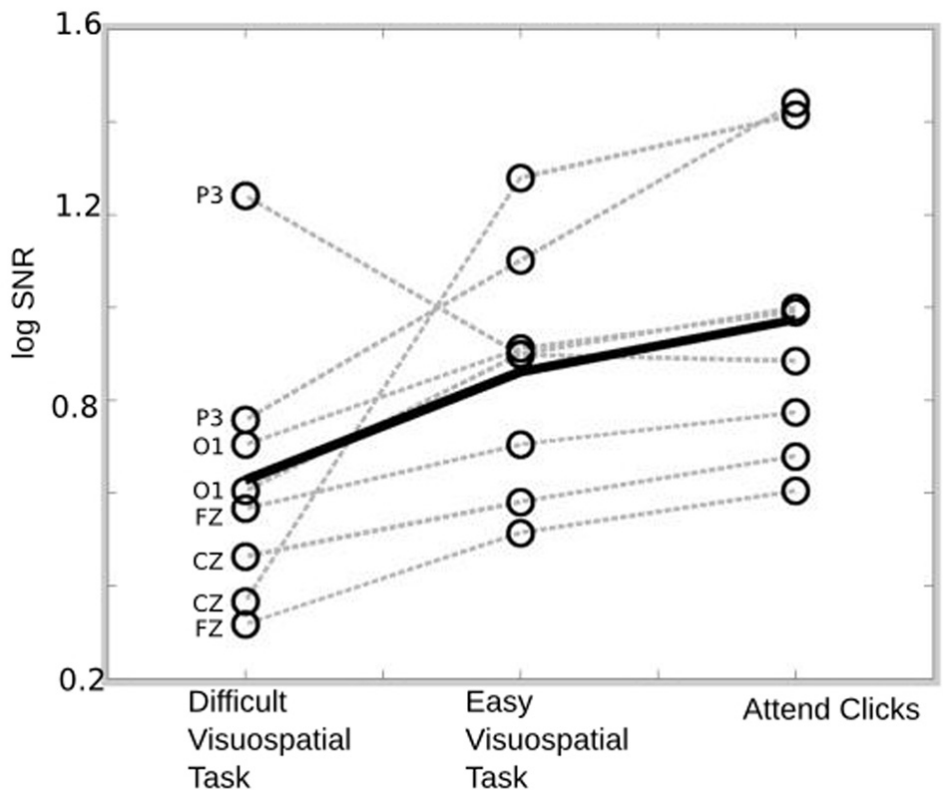
**SSAEP response for each condition**. The average 40 Hz response is indicated for each subject and condition (empty circles). The overall average response is indicated by the solid black line and the pattern of individual responses across conditions are indicated by the dotted gray lines. The response to the 40 Hz stimulus is largest when individuals attend to the stimulus, followed by when they perform the easy visuospatial task, followed by the difficult visuospatial task.

We examined whether auditory responses differed when individuals attended to modulations in the auditory stimuli, when they ignored the stimulus and performed an easy visuospatial task, or when they ignored the stimulus and performed a difficult visuospatial task. The log SNR of the 40 Hz auditory responses are indicated in Figure 3 for each of the three conditions. Seven out of eight individuals demonstrate a monotonic increase in response to the stimulus going from the difficult task to the easy task to “attend clicks.” This pattern is present in the overall average (solid black lines), and is represented statistically by a within-subject One-Way ANOVA indicating significant differences in log SNR across the three tasks [*F*_(2, 14)_ = 5.09, *p* = 0.0218]. No significant differences were observed when the log amplitude of the 40 Hz response was used as the dependent measure [*F*_(2, 14)_ = 0.21, *p* = 0.8109] or when the average log amplitude of the surrounding bins (i.e., the noise) was used as a dependent measure [*F*_(2, 14)_ = 0.24, *p* = 0.2096].

**Figure 4:**
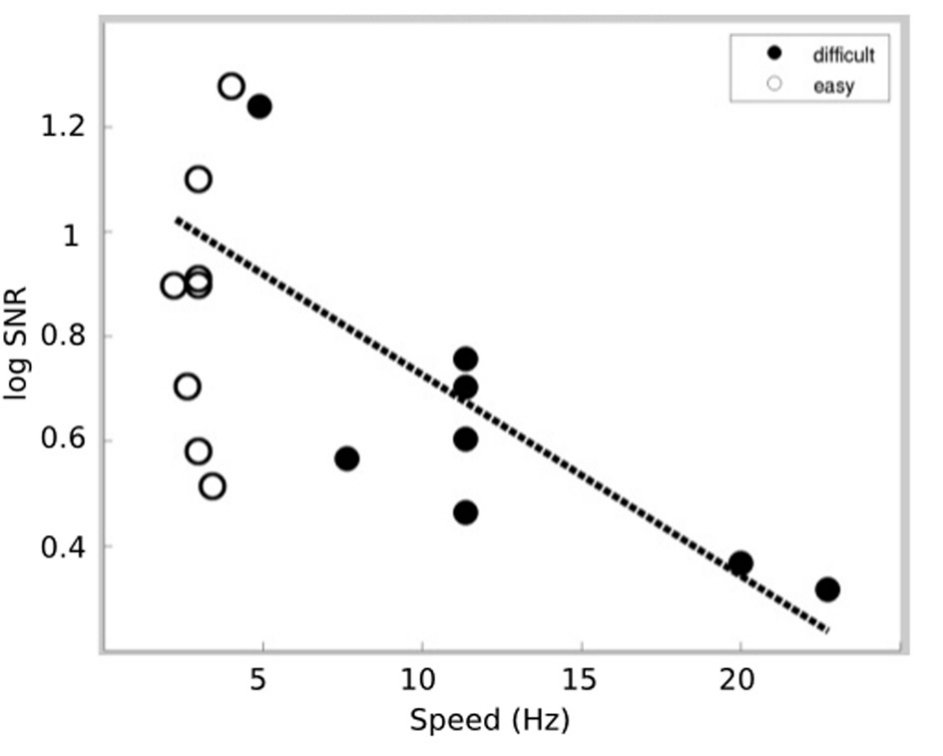
**SSAEP responses for each subject as a function of the speed**. The responses for the difficult task are indicated by the filled circles while responses for the easy task are indicated by the empty circles. In general, we find that reduced SSAEP responses correspond to increasing speed (i.e., difficulty) in the visuospatial task. The dotted line indicates the linear fit to the data within the difficult condition.

The relationship between auditory responses and task difficulty was further explored by examining the correlation between the log SNR of the 40 Hz response and the speed of the Tetris pieces. Our hypothesis was that the increased speed (e.g., increased difficulty) of Tetris would increase visuospatial resources at the expense of auditory processing. Thus, increased difficulty within the Tetris task is expected to be associated with reduced cortical responses at 40 Hz. This pattern is demonstrated in Figure 4 by plotting the log SNR against speed (Hz) for both the easy and difficult Tetris tasks. The figure demonstrates a negative relationship between log SNR and speed (Hz), which is represented by a significant negative correlation both when the results from the easy and difficult conditions were combined (*r* = −0.64; *p* = 0.0066) as well as separately within the difficult results (*r* = −0.79; *p* = 0.0209).

## Discussion

Our results demonstrate that cortical responses to an auditory stimulus are influenced by an individual's goal-directed attention to the stimulus, and by the degree of difficulty of a visuospatial task. Importantly, we have shown that increases in the speed of the visuospatial task are correlated with decreases in the cortical response to the 40 Hz auditory stimulus. These findings are consistent with the theory that increases in visuospatial difficulty result in increased utilization of higher order resources toward the visuospatial task, and reduced higher order resources toward processing information within sensory modalities that are not relevant to the task (Hein et al., 2007).

Auditory cortical responses were isolated in the current study by examining EEG responses to a 40 Hz auditory stimulus. An advantage of this approach is that the steady state response (i.e., the signal) is concentrated within the narrow frequency of the stimulus, while spectral changes due to muscle and eye artifacts (i.e., noise) will be distributed (Silberstein, 1995). Interestingly, we found differences in the signal-to-noise ratio across conditions, but no significant differences in the noise (e.g., 38 and 42 Hz) across conditions. This finding demonstrates that the results are due to differences in the normalized 40 Hz response, but not broadband changes in spectral amplitude that may result due to artifacts or changes in the spectral baseline.

The following additional points should be considered when interpreting the results. First, it is likely that some individuals contain greater prior experience with the task than others. While, individual video game experience was not collected, it is likely that increased video game experience would be associated with better performance within the Tetris task, which is reflected in the speed at which the Tetris pieces fell. Thus, the finding of a negative correlation between auditory responses (e.g., log SNR) and the speed of the pieces (Figure 4) is consistent with the theory that individuals with greater expertise were better able to focus on the visuospatial task and to ignore or suppress the irrelevant auditory stimuli. An additional consideration for further studies is whether the brain networks that modulate suppressing irrelevant sensory modalities may overlap with the brain networks that facilitate enhancing information across modalities (e.g., with multisensory integration). In addition, the present results were obtained within a population of males, which can limit the generalizability of the findings.

Auditory processing was examined within the current study by measuring EEG responses to a 40 Hz auditory stimulus. In this context, it is important to note that different sensory systems can be sensitive (i.e., generate larger responses) to different input frequencies (Regan, 1989), and different input frequencies can target brain networks with functionally distinct properties (Ding et al., 2006; Bridwell and Srinivasan, 2012). The auditory system demonstrates a 40 Hz transient response to tones (Tiitinen et al., 1993), suggesting that this frequency represents the intrinsic oscillatory frequency of auditory processing. This intrinsic nature of auditory 40 Hz responses is well supported by the finding that frequency tagged auditory responses peak at 40 Hz (Galambos et al., 1981; Picton et al., 2003), suggesting that 40 Hz auditory inputs may entrain and reveal the functional properties of the auditory system (Basar, 1999). This motivates its use in examining auditory cortical modulations with attention (Ross et al., 2004), and auditory/visual attention (Saupe et al., 2009; Keitel et al., 2011). However, it should be noted that while the auditory response peaks at 40 Hz, enhanced responses may also be observed within neighboring gamma-band frequencies (Galambos et al., 1981), and these input frequencies could potentially reveal similar modulations with attention.

Previous studies have examined the influence of visual load on the N1 and P2 ERP components (Regenbogen et al., 2012), as well as with the mismatch negativity (MMN) (Yucel et al., 2005; Haroush et al., 2010). Early evoked potentials, such as the N1, are thought to represent basic sensory processing, and these responses may be measured after averaging over repeated presentations of a basic auditory stimulus (Näätänen et al., 2011). The MMN, in contrast, represents the average response to deviant auditory tones that are presented within a series of more frequent auditory tones. In order to “detect” rare events, the brain must maintain a statistical representation or expectation of auditory stimuli, such that deviations from this expectation result in a reorientation of attention to the rare event. The MMN is therefore sensitive to more complex aspects of the auditory environment, and tends to demonstrate greater sensitivity to attentional manipulation (May and Tiitinen, 2010).

The different sensitivities of these evoked potentials to attention and early sensory processing suggests that they may demonstrate different sensitivity to visual load. Modulations within early evoked responses with visual load suggest the influence of visual load on early sensory processing, while modulations within the MMN demonstrate the influence of visual load on the auditory sensitivity to rare events (Haroush et al., 2010; Näätänen et al., 2011). This reflects a more complex level of auditory processing, which would potentially be disrupted to a greater degree when overlapping brain areas are utilized within visuocognitive tasks (Yucel et al., 2005). The frequency tagged auditory responses in the current study likely reflect the combination of the early auditory cortical responses as well as their integration with higher order brain areas. Thus, the frequency tagged response in our study may potentially reflect the interplay between early sensory areas and their modulation by higher level brain areas, such as the frontal cortex.

The influence of visual load has primarily been examined on auditory responses to rare tones (e.g., represented by the MMN) (Haroush et al., 2010). The influence of visual load on gamma (i.e., 40 Hz) responses was demonstrated by Tiitinen et al. (1993) with a similar experimental paradigm as the present study. Their study indicates that the 40 Hz auditory transient responses increase (compared to reading) when individuals passively ignore the tones, and further increase when they attend to the tones. An important distinction, however, is that the 40 Hz responses were elicited transiently from a brief auditory tone, while the 40 Hz responses in the current study were targeted by isolating frequency tagged 40 Hz cortical responses. A potential advantage of the frequency tagging approach is that the 40 Hz responses are robust to noise and can be distinguished from broadband increases in power that arise due to muscle tension or movement. Thus, the current approach may be useful for tracking an individual's attentional engagement within subsequent mobile EEG studies and brain-computer interface (BCI) based interventions.

An important distinction among previous studies is the degree of engagement and complexity of the visual task. For example, Tetris draws upon visuospatial resources (Sims and Mayer, 2002), reading draws upon visuocognitive processing (Welcome and Joanisse, 2012), and the visuomotor task in Yucel et al. (2005) encourages rapid visuomotor feedback and integration. Each of these studies demonstrate modulations in auditory responses with increases in task difficulty, but a series of simple detection and discrimination tasks have been unable to generate similar findings (for review see Haroush et al., 2010). These findings highlight the importance in considering the perceptual and cognitive resources required within a given task when examining its influence on attentional load (Lavie, 2010). Differences in task complexity and engagement could account in part for the inability of some studies to detect differences in auditory responses with increasing visual load. For example, simple detection and discrimination tasks may place an emphasis on automatic early visual processing without engaging higher level brain areas to the same extent as more complex tasks. While perceptual load within one modality appears to influence responses to irrelevant stimuli within the same modality (Lavie et al., 2004), there appears to be less of an influence of perceptual load within one modality on irrelevant sensory responses within another modality (Talsma et al., 2006).

Similar distinctions have been demonstrated within the load theory of attention and cognitive control (Lavie, 2005, 2010). For example, it has been demonstrated that there is reduced interference from an irrelevant distracter when individuals perform a high perceptual load task, but an increased interference from visual distracters when individuals perform a high memory load task. This finding suggests that an increased memory load is accompanied by a reduction in cognitive control resources that are available for suppressing irrelevant distracters. Within the context of the current study, individuals likely recruit the complex array of brain networks that facilitate visuocognitive processing during Tetris. These brain networks likely overlapped with the brain networks involved in processing the irrelevant auditory stimulus, resulting in a reduced 40 Hz auditory responses.

## Conclusion

This study investigated the influence of visuospatial attention on 40 Hz auditory cortical responses. EEG responses were measured to a 40 Hz auditory stimulus while individuals attended to modulations in the amplitude of the 40 Hz stimulus, and as a function of the difficulty of the visuospatial task Tetris. The results demonstrate the influence of visuocognitive demands on the sensory processing of irrelevant auditory stimuli. We found significant differences in the log SNR of the 40 Hz responses across the three conditions, demonstrating the influence of attention on the auditory 40 Hz response. Importantly, the log SNR of the 40 Hz response was significantly correlated with the speed (i.e., difficulty) of the task, indicating that auditory responses are reduced with increasing visuospatial load. Overall, the results demonstrate that 40 Hz auditory responses are influenced by an individual's goal-directed attention to the stimulus, and by the degree of difficulty of a complex visuospatial task.

### Conflict of interest statement

The authors declare that the research was conducted in the absence of any commercial or financial relationships that could be construed as a potential conflict of interest.
